# Self-medication practice with anti-malarial drugs and its associated factors among patients with fever attending public health facilities in Dera District, Northwest Ethiopia

**DOI:** 10.1371/journal.pone.0352762

**Published:** 2026-07-06

**Authors:** Wondimnew Desalegn Addis, Agmas Wassie Abate, Negede Yiheyis Tasew, Amare Yirga Abate, Belete Asnake Sitotaw, Abay Semagn Kassie, Elelta Mulugeta Ayalew, Birhanemaskal Malkamu, Kindie Mitiku

**Affiliations:** 1 Public and Environmental Health Education and Service Directorate, Department of Epidemiology, College of Health Sciences, Debre Tabor University, Debre Tabor, Ethiopia; 2 School of Medicine, Department of Integrated community and Mental Health, College of Health Sciences, Debre Tabor University, Debre Tabor, Ethiopia; 3 South Gondar Zone Health Department, Debre Tabor, Ethiopia; 4 Department of Pediatrics, Medical Doctor and Pediatrician, Debre Tabor Comprehensive Specialized Hospital, Debre Tabor, Ethiopia; 5 Public and Environmental Health Education and Service Directorate, Department of Health Informatics, College of Health Sciences, Debre Tabor University, Debre Tabor, Ethiopia; 6 Department of Medical Laboratory Sciences, College of Health Sciences, Debre Tabor University, Debre Tabor, Ethiopia; 7 Public and Environmental Health Education and Service Directorate, Department of Reproductive Health, College of Health Sciences, Debre Tabor University, Debre Tabor, Ethiopia; Bule Hora University, ETHIOPIA

## Abstract

**Background:**

The issue of self-medication with anti-malarial drugs is a critical public health challenge. Despite national guidelines promoting diagnostic-confirmed treatment, evidence addressing the prevalence of self-medication with anti-malarial drugs and drivers in Ethiopia is scarce. Therefore, this study aimed to bridge this gap and to provide meaningful insights into ongoing efforts to curb the misuse of anti-malarial drugs and helping the strategy of malaria eliminationby investigating the prevalence of self-medication with anti-malaria drugs and its associated factors.

**Methods:**

An institution-based cross-sectional study was conducted in Dera District, Northwest Ethiopia, from June 1–30, 2025. A total of 591 febrile patients were selected using a stratified multistage sampling technique. Data were collected through interviewer-administered structured questionnaires developed by reviewing different related literatures. Data entry and analysis were done using Epi-Data version 4.6 and STATA 17. Multivariable binary logistic regression analysis was performed to identify factors associated with self-medication. Statistical significance was declared at a p-value less than 0.05.

**Result:**

The prevalence of self-medication with anti-malarial drugs was 42.8% (95% CI: 38.9%–46.8%). Factors positively associated with self-medication included: monthly income of ≥5000 ETB (AOR = 1.64, 95% CI: 1.04–2.59), poor knowledge about malaria (AOR = 1.68, 95% CI: 1.12–2.55), poor risk perception towards self-medication (AOR = 2.10, 95% CI: 1.40–3.14), and distance ≤5 km from private drug sellers (AOR = 1.65, 95% CI: 1.10–2.47). Community-based health insurance membership was negatively associated with self-medication (AOR = 0.60, 95% CI: 0.41–0.88).

**Conclusion:**

Self-medication with anti-malarial drugs was moderate (33.4%_66.6%) in the study area based on percentile classification. The findings highlighted the need for designing different strategies to reduce inappropriate drug use and promoting community-based health insurance enrollment.

## 1 Introduction

Malaria is a life-threatening parasitic disease caused by *Plasmodium species* and transmitted by the infected female Anopheles mosquitoes [1,2]. It remains a major health challenge; elimination efforts are hampered by weak diagnostic coverage, anti-malarial resistance, and widespread self-treatment practices [3,4]. Self-medication is defined as the use of medicines without the advice or prescription of healthcare professionals [5]. In malaria, this often involves taking anti-malarial drugs such as Artemisinin-based Combination Therapies (ACTs), chloroquine, or quinine without prior diagnostic testing [6,7]. Contributing factors include perceived familiarity with malaria symptoms, financial constraints, poor access to health facilities, and the ready availability of drugs from pharmacies, shops, or informal vendors [8–10].

Self-medication with anti-malarial drugsdelays the correct diagnosis of other febrile illnesses, increases the risk of complications, and sustains malaria transmission within communities [11]. Critically, inappropriate and incomplete treatment contributes to the development of drug-resistant parasites. Artemisinin resistance, characterized by delayed parasite clearance and linked to molecular mutations, has already been detected in parts of Africa, with early warning signals emerging from Ethiopia [12–14].

Previous studies in Africa and Asia report highly variable prevalence of anti-malarial self-medication, ranging from 25% to more than 70% [9,15–19]. In Ethiopia, community-based studies have found prevalence between 17.8% and 64.5% [10,20]. Factors associated with self-medication include age, sex, education, occupation, household income, distance to health facilities, and knowledge of malaria treatment [10,19,21–24]. However, most existing evidences come from community surveys that is a general study which may not capture patients already presenting with fever at health facilities specifically.

The Dera district is among the highest malaria-reporting areas in Ethiopia, with repeated outbreaks and reports of unprescribed anti-malarial use [14,25]. Yet, little is known about the magnitude and determinants of this practice in patients attending health facilities.Therefore, this study aimed to assess the prevalence of self-medication with anti-malarial drugs and associated factors among febrile patients attending public health facilities in Dera District, Northwest Ethiopia. Generating such evidence is essential to inform targeted interventions, improve rational drug use, and support Ethiopia’s malaria elimination strategies.

## 2 Methods and materials

### 2.1 Study design, setting, and period

An institution-based cross-sectional study was conducted from June 1–30, 2025. This study was conducted in the Dera district of the Amhara Region, Ethiopia. The district is located 611 km from Addis Ababa, the capital city of Ethiopia, and 47 km Northwest of Bahir Dar, the regional capital. Dera covers an area of approximately 1,474 km² with a total population of 284,701, of whom 261,231 are rural dwellers. Administratively, the district is divided into 42 kebeles (39 rural and 3 urban), all of which are classified as malarious. The district has 51 governmental health facilities (11 health centers, 39 health posts, and one primary hospital) and 12 private clinics (2 medium and 10 lower-level clinics). Despite the heavy malaria burden, health facilities frequently face challenges such as stock outs of essential medicines, which may lead patients to seek alternative treatment options.

### 2.2 Population and eligibility criteria of the study

The source population for this study was all febrile patients visiting public health facilities in Dera District. The study population consisted of febrile patients who attended the randomly selected public health facilities during the data collection period.

Febrile patients aged 18 years or above (self report) were included in the study, since adults are more likely to make independent decisions on self-medication and provide reliable self-reported data. Patients who were not permanent residents of the district (living there for less than six months) or who were unable to respond due to serious illness were excluded to ensure accurate representation of the local population and data quality.

### 2.3 Sample size determination and sampling procedure

The minimum required sample size was calculated by considering both objectives. For the first objective, a single population proportion formula was applied with a 95% confidence level, 5% margin of error, and a prevalence of self-medication of 37.3% from a study in southwest Ethiopia [10]. The initial sample size was 360, which, after applying a design effect of 1.5 and a 10% non-response adjustment, gave a final sample size of 600. For the second objective, calculations using EPI INFO Stat Calc produced smaller sample sizes for various factors (Table 1), so the larger sample size of 600 was taken as the minimum required.

**Table 1:** Sample size calculation using EPI_INFO StatCalc considering factors affecting self-medication with anti-malarial drugs among patients with fever at public health facilities in Dera District, northwest Ethiopia, 2025.

Exposure variable	CI	Power	The ratio of Unexposed to exposed	% of outcomes in Unexposed	OR	%of outcome in Exposed	Sample size including 1.5 design effect & 10% contingency	Reference
Keep drugs at home	95	80	3.1	67.2	0.27	35.6	197	[26]
Information on Malaria treatment	95	80	2.3	52	4.33	82.3	174	[27]
Perceived the risk of self-medication	95	80	2.5	47.3	0.26	18.7	139	[8]

**Fig 1:**
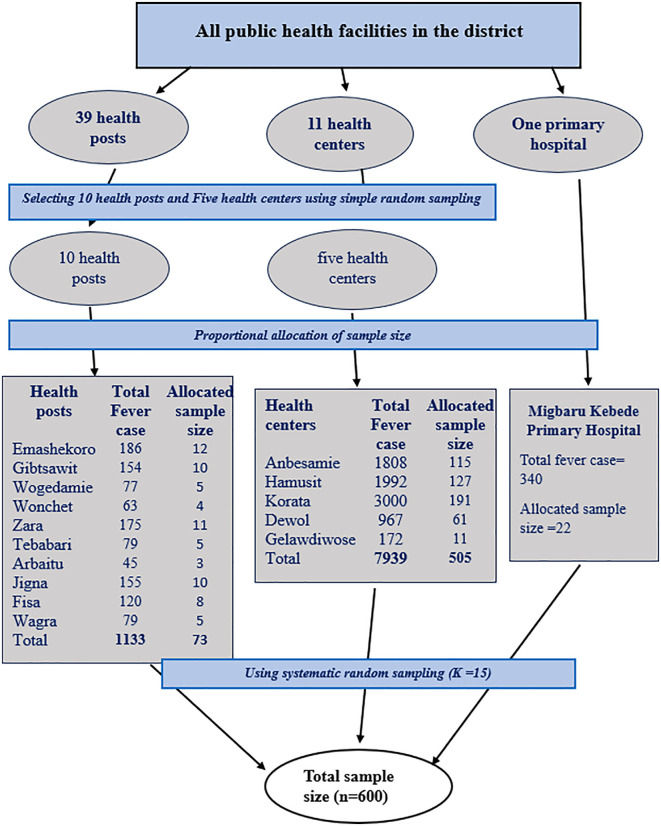
Schematic presentation of sampling procedure to select study participants among patients with fever attending public health facilities in Dera District, Northwest Ethiopia, 2025.

A multi-stage sampling technique was used to select participants. From 51 governmental health facilities (11 health centers, 39 health posts, and 1 primary hospital), 5 health centers and 10 health posts were randomly chosen, while the hospital was included purposively. The sample was proportionally allocated to each selected facility based on the number of fever cases reported during the same month of the previous year. Finally, systematic random sampling was employed to select patients. Using the total expected number of fever cases (9,412) and the final sample size (600), the sampling interval (k) was calculated as 15. A random starting point between 1 and 15 was chosen, and every 15th febrile patient was interviewed until the allocated sample size for each facility was reached; During selection the random start was 3 so 3, 18, 33…900 were included; for instance at Anbesamie Health Centre febrile patients arriving at the institution for service orderly 3,18,33…1725 were included to the study (Fig 1).

### 2.4 Variables of the study

The dependent variable of this study was self-medication practice with anti-malarial drugs, measured as “Yes or No”. The independent variables were categorized into three groups. Socio-economic factors included age, sex, marital status, educational status, occupation, monthly income, family size, and place of residence. Health service-related factors included community-based health insurance (CBHI) enrollment status, distance to the nearest health facility, distance to a drug shop, exposure to malaria treatment, cost affordability, and history of drug stock out exposure. Individual knowledge and attitude factors included knowledge about malaria, knowledge about self-medication, and risk perception toward self-medication.

### 2.5 Operational definitions

**Self-medication practice with anti-malarial drugs:** Respondents were assessed through a set of Likert scale item questions, with a response ranging from 1 to five (strongly disagree to strongly agree). The total mean practice score was computed, and respondents who scored a mean or above were considered as practicing self-medication with anti-malarial drugs, and those who scored below the mean were considered as not practicing anti-malarial drug self-medication [10].

**Knowledge about malaria:** Respondents were asked a knowledge question about malaria, the correct answer was coded as “1”, and the incorrect answer was coded as “0”. Then the mean knowledge score was computed, and those who had a score of the mean value or above were considered as knowledgeable, and those below the mean were considered as not knowledgeable [26].

**Knowledge about self-medication:** Respondents were asked a knowledge question about self-medication; the correct answer was coded as “1”, and the incorrect answer was coded as “0”. Then the mean knowledge score was computed, and those who had a score of the mean value or above were considered as knowledgeable, and those below the mean were considered as not knowledgeable [10].

**Risk perception towards self-medication:** Respondents were interviewed using a Likert scale item with five response categories (0 = strongly disagree, 1 = disagree, 2 = neutral, 3 = agree, 4 = strongly agree). The highest score was calculated by multiplying the highest scale value by the total number of questions. An individual was considered to have “perceived the risk” if they had at least 75% agreement; otherwise, “Not perceived the risk.” [22].

### 2.6 Data collection tool and procedure

The data were collected using an interviewer-administered structured questionnaire, which was developed by reviewing different literatures [8,10,19,21,22,27]. The tool was designed to collect data on Socio-demographic factors, service-related factors, patient knowledge, and risk perception about malaria, and anti-malarial self-medication practice. It was prepared in English, and then translated into Amharic for data collection purposes, and then back-translated to English by subject matter experts to ensure the meaning is retained. Then the interviews were conducted at the existing sites while they completed all the services at the health facilities. The data were collected by five BSc. nurses under the supervision of two public health officers who were trained on the principles of data collection, components of tools, and ethical issues of the study.

### 2.7 Data quality control

The data quality for this study was maintained through a careful design of the tools used for the data collection. Before data collection, data collectors were given a one-day training session to ensure a common understanding of the objectives, tools, and processes involved in data collection. The tool was pretested on 5% (30 patients with fever) of the sample size before the actual data collection at public health facilities in Fogera district, Ethiopia. The internal reliability of the tools was checked using Cronbach’s alpha value (>0.7). Besides, supervisors and the primary investigator evaluated the collected data for consistency, clarity, accuracy, and completeness regularly. To differentiate the type of drug used for self-medication without recall bias, data collectors had anti-malarial drugs during data collection to select the drug they used for self-medication and asked whether they had taken the drugs within a year. To avoid repeated data collection when participants may visit health facilities more than twice during data collection respondents were asked whether they had been asked about self medication study that month or not since the data collection period was only one month. If they said “yes we had been asked” the data collectors skipped them.

### 2.8 Data processing and analysis

After data collection, it was entered, cleaned, and coded using Epi-Data version 4.6, and then exported to STATA 17 for analysis. Descriptive statistics were used to summarize the data through simple frequency tables and figures. Binary logistic regression was used to identify factors associated with self-medication with anti-malarial drugs among patients with fever. All variables with a p-value <0.25 in the bivariable analysis were included in the multivariable analysis to account for potential confounders. The model’s fitness was evaluated using the Hosmer-Lemeshow test,whichyielded a p-value of >0.05. Before running multi-variable regression, multi-co linearity was checked using the variance inflation factor (VIF), which was < 10 for all variables. Odds ratios with 95% confidence intervals were computed to quantify the strength of associations between factors and the self-medication practice. Statistical significance was determined at p-values less than 0.05.

### 2.9 Ethical consideration

Following approval of the proposal, ethical clearance and a formal permission letter were obtained from Debre Tabor University College of Health Sciences (DTU/REF/379/17). Verbal informed consent was obtained to respect participants’ comfort and cultural preferences, ensuring that consent was given freely without the potential barriers or concerns sometimes associated with signing written documents. Before data collection, trained data collectors explained the study’s purpose, procedures, risks, and benefits clearly in Amharic Language. Participants were allowed to ask questions and voluntarily decide whether to participate or not and was recorded on each questionairre. Participants were assured that their participation was entirely voluntary, their responses were kept confidential and anonymous, and no personal identifiers were recorded. No compensation was provided for participation.

## 3 Results

### 3.1 Socio-demographic characteristics of respondents

In this study, a total of 591 patients with fever attending public health facilities in Dera District were included, with a response rate of 98.5%. The mean age of the respondents was 38 (SD ± 11.7) years, and about 191 (32.3%) were in the 35–44 age group. Among the total respondents, 342 (57.9%) were males. In terms of marital status, 392 (66.3%) were married. Concerning educational status, 367 (62.1%) had no formal education. Concerning occupation, 497 (84.3%) were farmers. For household monthly income, 224 (37.9%) earned between 2000 and 4999 ETB. About household family size, 406 (68.7%) lived in households with five or fewer members. Finally, 431 (72.9%) of the respondents resided in rural areas (Table 2).

**Table 2:** Socio-demographic characteristics of respondents in a study assessing the practice of self-medication with anti-malarial drugs among patients with fever in Dera District, Northwest Ethiopia, 2025.

Variables	Response categories	Frequency	Percentage
Sex	Male	342	57.9
	Female	249	42.1
Age	18-24	84	14.2
	25-34	142	24.0
	35-44	191	32.3
	≥45	174	29.4
Marital status	Never married	124	21.0
	Married	392	66.3
	Divorced	44	7.4
	Widowed	31	5.2
Educational status	No formal education	367	62.1
	Primary education	141	23.9
	Secondary school	38	6.4
	College and above	45	7.6
Occupation	Farmer	497	84.1
	Merchant	57	9.6
	Employe	21	3.6
	Others	16	2.7
HH’s monthly income (ETB)	<2000	185	31.3
	2000-4999	224	37.9
	≥ 5000	182	30.8
HH’s family size	≤ 5	406	68.7
	>5	185	31.3
Residency	Urban	160	27.1
	Rural	431	72.9

### 3.2 Health service-related factors

Out of the total respondents, 403 (68.2%) reported being members of the community-based health insurance (CBHI) scheme. In terms of distance from a public health facility, 357 (60.4%) resided within five Km. Regarding proximity to private drug sellers, 382 (64.6%) lived within five kilometers. A history of confirmed malaria illness was reported by 457 (77.3%) participants. Concerning recent use of public health services, 542 (91.7%) had visited a public health facility. Challenges at public health facilities were reported by 438 (74.1%) respondents. Among the specific challenges faced, 346 (79.0%) mentioned long waiting times, 135 (30.8%) cited high treatment costs, 274 (62.6%) experienced medication stockouts, 57 (13.0%) expressed a lack of trust in health workers, and 116 (26.5%) reported transport-related issues (Table 3).

**Table 3:** Health service-related factors associated with the practice of self-medication with anti-malarial drugs among patients with fever in Dera District, Northwest Ethiopia, 2025.

Variables	Response categories	Frequency	Percentage
CBHI membership status	Yes	403	68.2
	No	188	31.8
Distance from public health facility	≤ 5 Km	357	60.4
	>5 Km	234	39.6
Distance from private drug sellers	≤ 5 Km	382	64.6
	>5 Km	209	35.4
History of confirmed malaria illness	Yes	457	77.3
	No	134	22.7
History of public health facility visit	Yes	542	91.7
	No	49	8.3
Challenges faced at public health facilities	Yes	438	74.1
	No	104	17.6
Type of challenges faced	Long waiting time	346	79.0
	High cost of treatment	135	30.8
	Medication stockout	274	62.6
	Lack of trust in Health workers	57	13.0
	Lack of transport	116	26.5

### 3.3 Knowledge about malaria

Among the total respondents, 583 (98.6%) had heard about malaria. The most frequently mentioned source of information was health workers, cited by 534 (90.4%) participants. Regarding knowledge of common signs and symptoms, 522 (88.3%) identified fever as a symptom. When asked whether fever is always due to malaria, 485 (82.1%) responded affirmatively. Concerning malaria transmission, 324 (54.8%) correctly identified mosquito bites as the mode of transmission. In terms of prevention, 553 (93.6%) mentioned the use of insecticide-treated bed nets. The belief that malaria is a fatal disease was held by 535 (90.5%) respondents, while 544 (92.0%) acknowledged that malaria can affect people of all ages. Overall, 415 (70.2%) of the participants demonstrated good knowledge about malaria (Table 4).

**Table 4:** Knowledge about malaria among patients with fever in a study assessing self-medication practice with anti-malarial drugs in Dera District, Northwest Ethiopia, 2025.

Knowledge question	Response	Frequency	Percentage
Heard about malaria	Yes	583	98.6
	No	8	1.4
Source of information	Mass media	141	23.9
	Health workers	534	90.4
	Local authorities	232	39.3
	Others source	10	1.7
Common signs and Symptoms of malaria	Fever	522	88.3
	Headache	447	75.6
	Shivering	451	76.3
	Joint pain	325	55.0
Fever is always due to malaria	Yes	485	82.1
	No	106	17.9
Mode of malaria transmission	Mosquito bite	324	54.8
	Drinking dirty water	95	16.1
	Exposed to the sun	47	8.0
	Exposed to cold air	121	20.5
	Contact with malaria patients	4	0.7
Malaria prevention methods	Using an insect net-treated bed net	553	93.6
	Larvae source management	379	64.1
	Indoor residual spray	373	63.1
	Early diagnosis and treatment	284	48.1
Malaria is a fatal disease	Yes	535	90.5
	No	56	9.5
Malaria affects people of all ages	Yes	544	92.0
	No	47	8.0
**Overall knowledge about malaria**	**Poor**	176	29.8
	**Good**	415	70.2

### 3.4 Knowledge about self-medication with anti-malarial drugs

Out of the total respondents, 432 (73.1%) correctly identified self-medication as taking drugs without consulting health care workers, followed by taking leftover anti-malarial drugs 375 (63.5%), using drugs through friend counseling 258 (43.7%), and taking it without a prescription 266 (45.0%). Regarding the right sources of anti-malarial drugs, 539 (91.2%) mentioned public health facilities, 373 (63.1%) drug stores with prescriptions, 196 (33.2%) shops, and 103 (17.4%) leftover drugs from another family. Concerning the risks of self-medication, drug resistance was reported by 420 (71.1%), taking an incorrect dose by 418 (70.7%), unmanageable side effects by 325 (55.0%), masking of other diseases due to fever by 323 (54.7%), and wastage of money by 290 (49.1%). Furthermore, 389 (65.8%) agreed that self-medication can lead to the spread of drug-resistant malaria. When asked who should decide to take anti-malarial drugs, the majority, 534 (90.4%), indicated health professionals, while 141 (23.9%) said themselves, 83 (14.0%) mentioned families or friends, and 38 (6.4%) chose others. Finally, the overall knowledge level revealed that 306 (51.8%) had poor knowledge about self-medication with anti-malarial drugs (Table 5).

**Table 5:** Knowledge about self-medication with anti-malarial drugs among patients with fever in Dera District, Northwest Ethiopia, 2025.

Knowledge questions	Frequency	Percentage
**What does self-medication with anti-malaria mean**		
Taking drugs without consulting HCWs	432	73.1
Taking leftover antimalarial drugs	375	63.5
Taking antimalarial drugs through friend counseling	258	43.7
Take an anti-malarial drug without a prescription	266	45.0
**The right source of anti-malarial drugs**		
Public health facilities	539	91.2
Drug stores with prescriptions	373	63.1
Shops	196	33.2
Leftover drug from another family	103	17.4
**What are the risks of self-medication?**		
Drug resistance	420	71.1
Taking the incorrect dose	418	70.7
Unmanageable side effects	325	55.0
Mask other diseases due to fever	323	54.7
Wastage of money	290	49.1
Others	31	5.2
**Self-medication with anti-malarial drugs can lead to the spread of drug-resistant malaria**		
Yes	389	65.8
No	202	34.2
**Who should decide to take anti-malaria drugs?**		
Health professionals	534	90.4
My self	141	23.9
Families or friends	83	14.0
Others	38	6.4
**Overall knowledge about self-medication**		
Poor	306	51.8
Good	285	48.2

### 3.5 Risk perception towards self-medication with anti-malarial drugs

Out of the total respondents, 128 (21.7%) strongly disagreed that it increases drug-resistant malaria, while 182 (30.8%) agreed and 69 (11.7%) strongly agreed with the statement. When asked whether taking anti-malarial drugs without health care workers (HCWs) might result in treating the wrong illness, 151 (25.5%) strongly disagreed, whereas 172 (29.1%) agreed, and 89 (15.1%) strongly agreed. Regarding potential side effects or allergies from self-medication, 113 (19.1%) strongly disagreed, 185 (31.3%) disagreed, and 114 (19.3%) strongly agreed. Concerning the risk of using the wrong dosage without HCW advice, 210 (35.5%) agreed and 122 (20.6%) strongly agreed, while 83 (14.0%) strongly disagreed. For the statement that self-medication may delay proper diagnosis and treatment, 235 (39.8%) agreed, while 119 (20.1%) strongly disagreed. Regarding the possibility that self-medication could mask a serious condition, 148 (25.0%) strongly disagreed and 114 (19.3%) strongly agreed. On the belief that using anti-malarial drugs without HCWs wastes money, 217 (36.7%) agreed and 105 (17.8%) strongly agreed. When asked about harmful drug interactions, 156 (26.4%) agreed and 134 (22.7%) strongly agreed, while 107 (18.1%) strongly disagreed. Overall, 406 (68.7%) of respondents perceived poor towards self-medication with anti-malarial drugs, while 185 (31.3%) had a good attitude (Table 6).

**Table 6:** Risk perception towards self-medication with anti-malarial drugs among patients with fever in Dera District, Northwest Ethiopia, 2025.

Question	Strongly disagree		Disagree		Neutral		Agree		Strongly agree	
	N	%	n	%	n	%	n	%	n	%
Self-medication increases drug-resistant malaria.	128	21.7	194	32.8	18	3.0	182	30.8	69	11.7
Taking antimalarials without HCWs may treat the wrong illness	151	25.5	137	23.2	42	7.1	172	29.1	89	15.1
Self-medication can cause serious side effects or allergies.	113	19.1	185	31.3	28	4.7	151	25.5	114	19.3
Using antimalarials without HCW advice risks the wrong dosage.	83	14.0	153	25.9	23	3.9	210	35.5	122	20.6
Self-medication may delay proper diagnosis and treatment.	119	20.1	140	23.7	23	3.9	235	39.8	74	12.5
Using antimalarials without HCW can mask a serious condition.	148	25.0	150	25.4	33	5.6	146	24.7	114	19.3
Using antimalarials without HCW wastes money	121	20.5	128	21.7	20	3.4	217	36.7	105	17.8
Using antimalarials without HCW causes harmful drug interactions.	107	18.1	177	29.9	17	2.9	156	26.4	134	22.7
**Overall attitude**	**Poor**		**406**				**68.7**			
	**Good**		**185**				**31.3**			

### 3.6 Self-medication practice with anti-malarial drugs

In this study, the prevalence of self-medication with anti-malarial drugs was 42.8% (95% CI: 38.9%–46.8%). Specifically, 152 (25.7%) agreed with the statement that they took anti-malarial drugs without consulting a health care worker. Additionally, 86 (14.6%) agreed with the idea of keeping anti-malarial drugs at home for future symptoms, indicating that the remaining majority may engage in this practice. A total of 119 (20.1%) agreed that they prefer self-medication to visiting a health facility for malaria. Regarding reuse, 162 (27.4%) agreed that they have reused leftover anti-malarial drugs for a fever. When asked about purchasing drugs without a prescription, 131 (22.2%) agreed with doing so. A total of 118 (20.0%) agreed that they take anti-malarial drugs based on advice from friends or family. Additionally, 128 (21.7%) agreed with using traditional healers or herbs instead of anti-malarial drugs. About 150 (25.4%) agreed that they take anti-malarial drugs based on past personal experience. Furthermore, 147 (24.9%) agreed with the following advice from non-medical people to take anti-malarial drugs. Concerning diagnostic practices, 164 (27.7%) agreed that they took anti-malarial drugs without confirming malaria with a test. Regarding treatment completion, 124 (21.0%) agreed that they would stop taking anti-malarial drugs when they feel better. A total of 138 (23.4%) agreed that they did not check the expiry date before self-medicating. Lastly, 140 (23.7%) agreed that they have shared anti-malarial drugs with others (Table 7).

**Table 7:** Self-medication practice with anti-malarial drugs among patients with fever in Dera District, Northwest Ethiopia, 2025.

Question	Strongly disagree		Disagree		Neutral		Agree		Strongly agree	
	N	%	N	%	n	%	n	%	n	%
I took anti-malarial drugs without consulting aHCW.	236	39.9	156	26.4	33	5.6	152	25.7	24	4.1
I keep anti-malarial drugs at home for future symptoms.	210	35.5	229	38.7	33	5.6	86	14.6	33	5.6
I prefer self-medication to visiting a health facility for malaria.	206	34.9	167	28.3	73	12.4	119	20.1	26	4.4
I’ve reused leftover anti-malarial drugs for a fever.	183	31.0	178	30.1	29	4.9	162	27.4	39	6.6
I buy anti-malarial drugs without a prescription.	248	42.0	118	20.0	53	9.0	131	22.2	44	7.4
I take anti-malarial drugs based on advice from friends or family.	182	30.8	202	34.2	53	9.0	118	20.0	36	6.1
I use traditional healers or herbs instead of anti-malarial	220	37.2	130	22.0	76	12.9	128	21.7	37	6.3
I take anti-malarial drugs based on past personal experience.	164	27.7	187	31.6	60	10.2	150	25.4	30	5.1
I follow advice from non-medical people to take anti-malarial drugs	201	34.0	134	22.7	65	11.0	147	24.9	44	7.4
I took anti-malarial without confirming malaria with a test.	154	26.1	177	29.9	63	10.7	164	27.7	33	5.6
I stop taking anti-malarial if I feel better.	238	40.3	98	16.6	77	13.0	124	21.0	54	9.1
I didn’t check the expiry date before self-medicating.	162	27.4	197	33.3	43	7.3	138	23.4	51	8.6
I’ve shared anti-malarial drugs with others.	170	28.8	163	27.6	50	8.5	140	23.7	68	11.5
**Overall practice**	**Yes**		**253**				**42.8**			
	**No**		**338**				**57.2**			

### 3.7 Factors associated with self-medication with anti-malarial drugs

Bi-variable binary logistic regression analysis was conducted to identify candidate variables for multivariable logistic regression, using a p-value threshold of 0.25. A total of fourteen variables met the criteria and were included in the final multivariable logistic regression model. In the final model, HHs’ monthly income, CBHI membership status, knowledge about malaria, and risk perception towards self-medication with anti-malarial drugs were found to be significantly associated with self-medication practice with anti-malarial drugs at a p-value less than 0.05.

Participants with a household monthly income of ≥5000 ETB were 1.64 times more likely to practice self-medication with anti-malarial drugs compared to those with an income of <2000 ETB (AOR = 1.64, 95% CI: 1.04–2.59, *p* = 0.034). Those who were members of CBHI were 40% less likely to self-medicate with anti-malarial drugs compared to non-members (AOR = 0.60, 95% CI: 0.41–0.88, *p* = 0.009). Participants who lived within 5 km of private drug sellers had 1.65 times higher odds of self-medicating with anti-malarial drugs compared to those who lived farther away (AOR = 1.65, 95% CI: 1.10–2.47, *p* = 0.014). Participants with poor knowledge about malaria were 1.68 times more likely to self-medicate with anti-malarial drugs compared to those with good knowledge, after adjusting for other variables (AOR = 1.68, 95% CI: 1.12–2.55, p = 0.014). Lastly, individuals with poor risk perception towards self-medication were 2.10 times more likely to practice self-medication with anti-malarial drugs than those with good risk perception (AOR = 2.10, 95% CI: 1.40–3.14, *p* < 0.001) (Table 8).

**Table 8:** Bi-variable and multivariable logistic regression analysis of factors associated with self-medication practice with anti-malarial drugs among patients with fever in Dera District, Northwest Ethiopia, 2025.

Variables	Self-medication practice with anti-malarial drugs		Crude OR (95% CI)	Adjusted OR (95% CI)	P-Value
	Yes (n = 253)	No (n = 338)			
**Sex**					
Male	137	205	0.77 (0.55-1.06)	0.85 (0.59-1.23)	0.392
Female	116	133	1.00	1.00	
**Age in years**					
18-24	47	37	1.72 (1.02-2.90)	1.55 (0.84-2.87)	0.159
25-34	54	88	0.83 (0.53-1.30)	0.77 (0.46-1.30)	0.326
35-44	78	113	0.93 (0.61-1.41)	0.87 (0.55-1.40)	0.578
≥45	74	100	1.00	1.00	
**Educational status**					
No formal education	170	197	1.00	1.00	
Primary education	51	90	0.66 (0.44-0.98)	0.82 (0.51-1.31)	0.408
Secondary school	16	22	0.84 (0.43-1.66)	0.72 (0.32-1.63)	0.432
College and above	16	29	0.64 (0.34-1.22)	0.69 (0.31-1.51)	0.350
**Occupation**					
Farmer	223	274	1.00	1.00	
Merchant	14	43	0.40 (0.21-0.75	0.59 (0.29-1.22)	0.158
Employe	8	23	0.76 (0.31-1.86)	1.44 (0.49 −4.18)	0.507
Others	8	8	1.23 (0.46-3.33)	1.92 (0.60-6.11)	0.271
**HH’s estimated monthly income in ETB**					
<2000	72	113	1.00	1.00	
2000-4999	96	128	1.18 (0.79-1.75)	1.26 (0.82-1.94)	0.298
≥ 5000	85	97	1.37 (0.91-2.08)	1.64 (1.04-2.59)	**0.034**
**Residency**					
Urban	56	104	1.00	1.00	
Rural	197	234	1.56 (1.07-2.28)	1.20 (0.77-1.89)	0.421
**CBHI membership status**					
Yes	158	245	0.63 (0.44-0.89)	0.60 (0.41-0.88)	**0.009**
No	95	93	1.00	1.00	
**Distance of the public health facility**					
≤ 5 Km	143	214	1.00	1.00	
>5 Km	110	124	1.33 (0.95-1.85)	1.20 (0.82-1.75)	0.345
**Distance from private drug sellers**					
≤ 5 Km	171	211	1.25 (0.89-1.77)	1.65 (1.10-2.47)	**0.014**
>5 Km	82	127	1.00	1.00	
**History of confirmed malaria**					
Yes	202	255	1.29 (0.87-1.91)	1.40 (0.88-2.24)	0.157
No	51	83	1.00	1.00	
**History of public health facility visit**					
Yes	236	306	1.45 (0.78-2.68)	1.68 (0.80-3.56)	0.171
No	17	32	1.00	0.00	
**Knowledge about malaria**					
Poor	92	84	1.73 (1.21-2.46)	1.68 (1.12-2.55)	**0.014**
Good	161	254	1.00	1.00	
**Knowledge about self-medication**					
Poor	145	161	1.48 (1.06-2.05)	1.41 (0.96-2.08)	0.079
Good	108	177	1.00	1.00	
**Risk perception towards self-medication with anti-malarial**					
Poor	200	206	2.42 (1.66-3.51)	2.10 (1.40-3.14)	**<0.001**
Good	53	132	1.00	1.00	

## 4 Discussions

This study aimed to assess the prevalence of self-medication with anti-malarial drugs and its associated factors, though the prevalence of self-medication with anti-malarial drugs was 42.8% (95% CI: 38.9%–46.8%). This finding is comparable to studies from Nigeria (42.7%) [28]. It can be justified by shared factors such as limited access to timely healthcare services, widespread availability of anti-malarial drugs without prescription in private clinics, similar levels of public awareness about the risks of self-medication, and common cultural practices of self-treatment. Additionally, in many malaria-endemic settings, individuals may rely on prior experience with malaria symptoms and treatment, leading to similar patterns of behavior across populations [29].

However, it is higher than reports from Sri Lanka (37% urban, 25% rural) [15], Asendabo, Ethiopia (37.3%) [10], and Northern Benin (38.81%) [19].The prevalence in this study is lower than findings from India (68.1%) [16], Nigeria (72%, 69%) [17,18]., Kigoma-Tanzania (69.6%) [24], Northwestern Tanzania (55.9%) [30], Western Kenya (60%) [31], Butajira, Ethiopia (64.5%) [20]. The variation in self-medication with anti-malarial drugs across studies can be attributed to differences in healthcare access, drug regulation, individual behaviors, and economic conditions. In settings where access to healthcare is limited, distances to health facilities are long, or drug shortages are frequent, self-medication tends to be more common [32].Additionally, this variation could also be due to seasonal or peak time of malaria in Dera district during the study period the month June is one the two peak malaria seasons in the area, the sensitivity of this practice to population characteristics, geographic location, and malaria transmission intensity that may over estimate the prevalence.

In this study, participants with a household monthly income of ≥5000 ETB were 1.64 times more likely to practice self-medication with anti-malarial drugs compared to those earning less than 2000 ETB. Similar findings have been reported in studies from Tanzania [9], Uganda [33] and Kenya [34], where higher-income groups were more likely to engage in self-medication due to greater purchasing power and access to private drug outlets. This finding suggests, individuals with more financial resources might prefer the convenience and immediacy of self-treatment, avoiding the time and costs associated with formal healthcare visits [8]. This emphasizes the need for targeted health education to promote safe medication practices across all income levels.

This study revealed that participants who were members of CBHI were 40% less likely to self-medicate with anti-malarial drugs compared to non-members. This suggests that health insurance membership may improve access to formal healthcare services by reducing financial barriers, encouraging individuals to seek professional diagnosis and treatment rather than resorting to self-medication. This finding was supported by the study done in Nigeria [32], Ghana [35], Tanzania [22], and Ethiopia [10], which showed that uninsured individuals were significantly more likely to self-medicate than insured individuals. Health insurance coverage has been associated with lower out-of-pocket expenses and increased use of formal health services, which in turn reduces the likelihood of inappropriate drug use [36,37].The finding highlights the important role of CBHI in promoting rational healthcare-seeking behavior and reducing the inappropriate use of anti-malarial drugs.

This study found that participants who lived within 5 kilometers of private drug sellers had 1.65 times higher odds of self-medicating with anti-malarial drugs compared to those who lived farther away. This finding was supported by the study report done in Uganda [33], in Tanzania [22], in Kasulu [24], and in Zambia [8]. This association may be explained by the ease of access and convenience offered by nearby drug outlets, which can encourage individuals to bypass formal health facilities and seek quick solutions to febrile illnesses [38]. Moreover, unlike public health facilities, private drug sellers usually do not require consultations, laboratory confirmation, or long waiting times.This finding implied that geographic proximity to drug outlets, while beneficial in terms of accessibility, may contribute to misuse of anti-malarial drugs, increasing the risk of drug resistance, delayed diagnosis of other illnesses, and ineffective treatment outcomes.

This study revealed that participants with poor knowledge about malaria were 1.68 times more likely to self-medicate with anti-malarial drugs compared to those with good knowledge. It is supported by the study done in rural Uganda [22] and in Parakou [19]. This can be justified by the reason that individuals with poor knowledge may misinterpret non-malarial febrile illnesses as malaria, leading to inappropriate drug use. Patients with inadequate understanding of malaria symptoms, transmission, and the importance of accurate diagnosis may lead individuals to treat themselves without seeking professional care. This highlights the need for improving community knowledge to reduce self-medication practices, promote timely care-seeking, and contribute to better malaria control and resistance prevention efforts.

Lastly, this study found that individuals with poor risk perception towards self-medication were 2.10 times more likely to self-medicate with anti-malarial drugs compared to those with good risk perception. Similar findings have been reported in studies from the Nodola district, Zambia [28], and Nigeria [39], where low risk perception was a significant predictor of self-medication. This finding emphasizes the urgent need for behavior change communication interventions that not only raise awareness but also actively reshape perceptions of risk associated with self-medication.

This study is one of the few analytical investigations specifically focused on self-medication with anti-malarial drugs among febrile patients attending public health facilities in a high-malaria-burden area. It was employed in a relatively large and representative sample size using a stratified multistage sampling technique, which enhances the representativeness of the findings. Despite its strengths, the cross-sectional nature of the study made it impossible to establish a temporal or causal relationship between the factors studied and the practice of self-medication with anti-malarial drugs. Secondly, the data were collected through interviews administered questions that are prone to social desirability bias may have influenced participants to underreport undesirable behaviors like self-medicationin which the true prevalence may increase from 42.8%, especially in a health facility setting, but the effect of this bias was minimal. Thirdly, the study only includes patients attending public health facilities. It misses individuals who self-medicate and do not seek care at public facilities, potentially underestimating the true prevalence of self-medication in the community. Lastly, findings from Dera District may not be directly generalizable to other districts of the region, due to variations in culture, healthcare access, economic conditions, and malaria epidemiology.

## 5 Conclusion and recommendation

This study revealed that self-medication with anti-malarial drugs is a moderatepractice (based on percentile classification: Low practice less than 33.3%, Moderate practice 33.4%_66.6% and High practice greater than 66.6%) among febrile patients in Dera districtinfluenced by factors such as higher household income, poor knowledge about malaria, poor risk perception, and proximity to private drug sellers, while community-based health insurance enrollment was protective. To address these different strategies had better to be designed, to minimize and avoid self medication practices. In addition, further qualitative research is recommended to better understand the underlying factors for self-medication and to design culturally appropriate interventions.

## Supporting information

S1 File SPSS data file Self Medication practice. (SAV)

## Abbreviations

ACTs: Artemisinin-based Combination Therapies
CBHI: Community-Based Health Insurance
CFR: Case Fatality Rate
RDTs: Rapid Diagnostic Tests
VIF: Variance Inflation Factor
Km: Kilometers

## References

[1] World Health Organization. WHO guidelines for malaria. Geneva: World Health Organization; 2023.

[2] World Health Organization. WHO guidelines for malaria. World Health Organization; 2022.

[3] World Health Organization. World malaria report 2023. World Health Organization; 2023.

[4] Akilimali A, Mufungizi I, Tague C, Rusho MA, Mugisha MJM, Karegeya A, et al. Supporting health systems of Ethiopia in the battle against malaria: a call for action. Ann Med Surg (Lond). 2025;87(1):432–5. doi: 10.1097/MS9.0000000000002799 40109631 PMC11918582

[5] Baracaldo-Santamaría D, Trujillo-Moreno MJ, Pérez-Acosta AM, Feliciano-Alfonso JE, Calderon-Ospina C-A, Soler F. Definition of self-medication: a scoping review. Ther Adv Drug Saf. 2022;13:20420986221127501. doi: 10.1177/20420986221127501 36211626 PMC9537481

[6] World Health Organization. WHO guideline on self-care interventions for health and well-being, 2022 revision. World Health Organization; 2022. 35914064

[7] World Health Organization. Global malaria programme operational strategy 2024-2030. Geneva: World Health Organization; 2024.

[8] Susiku NG, Jacobs C, Zgambo J, Kaonga P, Sitali-Zimba L. Determinants of self-treatment with antimalarials in Ndola district, Zambia: a cross-sectional study. medRxiv. 2024. doi: 2024.09.02.24312958

[9] Hertz JT, Madut DB, Tesha RA, William G, Simmons RA, Galson SW, et al. Self-medication with non-prescribed pharmaceutical agents in an area of low malaria transmission in northern Tanzania: a community-based survey. Trans R Soc Trop Med Hyg. 2019;113(4):183–8. doi: 10.1093/trstmh/try138 30597114 PMC6432801

[10] Kovalev V, Wells ML. Self-treatment practices for perceived symptoms of Malaria in Ethiopia. Cureus. 2020;12(7):e9359. doi: 10.7759/cureus.9359 32850230 PMC7444995

[11] Woldesenbet D, Tegegne Y, Mussema A, Tamene E, Mohamed K, Abebe W, et al. Can Ethiopia eliminate malaria? Malaria burden: insights from the pre-elimination era, current challenges and perspectives. Front Malar. 2025;3. doi: 10.3389/fmala.2025.1492444

[12] Balikagala B, Fukuda N, Ikeda M, Katuro OT, Tachibana S-I, Yamauchi M, et al. Evidence of artemisinin-resistant Malaria in Africa. N Engl J Med. 2021;385(13):1163–71. doi: 10.1056/NEJMoa2101746 34551228

[13] Alebachew M, Gelaye W, Abate MA, Sime H, Hailgiorgis H, Gidey B, et al. Therapeutic efficacy of pyronaridine-artesunate (Pyramax®) against uncomplicated Plasmodium falciparum infection at Hamusit Health Centre, Northwest Ethiopia. Malar J. 2023;22(1):186. doi: 10.1186/s12936-023-04618-y 37330475 PMC10276904

[14] Assefa A, Fola AA, Tasew G. Emergence of Plasmodium falciparum strains with artemisinin partial resistance in East Africa and the Horn of Africa: is there a need to panic?. Malar J. 2024;23(1):34. doi: 10.1186/s12936-024-04848-8 38273360 PMC10809756

[15] Wijesinghe PR, Jayakody RL, de A Seneviratne R. Prevalence and predictors of self-medication in a selected urban and rural district of Sri Lanka. WHO South East Asia J Public Health. 2012;1(1):28–41. doi: 10.4103/2224-3151.206911 28612776

[16] Rangari GM, Bhaisare RG, Korukonda V, Chaitanya YL, N H. Prevalence of self-medication in rural area of Andhra Pradesh. J Family Med Prim Care. 2020;9(6):2891–8. doi: 10.4103/jfmpc.jfmpc_204_20 32984145 PMC7491850

[17] Iribhogbe OI, Odoya EM. Self-medication practice with antimalarials & the determinants of malaria treatment-seeking behavior among postpartum mothers in a rural community in Nigeria. Pharmacoepidemiol Drug Saf. 2021;30(4):435–44. doi: 10.1002/pds.5178 33280184

[18] Bamikole OJ, Olajide TH, Adedeji BA, Ademola SA, Fayehun AF, Bukoye NO, et al. Drug use practices and self-treatment for suspected Malaria in Ibadan, Nigeria. Am J Trop Med Hyg. 2023;108(6):1122–6. doi: 10.4269/ajtmh.22-0489 37068754 PMC10540092

[19] Attinsounon CA, Sissinto Y, Avokpaho E, Alassani A, Sanni M, Zannou M. Self-medication practice against malaria and associated factors in the City of Parakou in Northern Benin: results of a population survey in 2017. AID. 2019;09(03):263–75. doi: 10.4236/aid.2019.93020

[20] Deressa W, Ali A, Enqusellassie F. Self-treatment of malaria in rural communities, Butajira, southern Ethiopia. Bull World Health Organ. 2003;81(4):261–8. 12764492 PMC2572446

[21] Isabella OA, Ann O, Oyeniyi AA, Ukamaka O. Knowledge and attitudinal dispositions as factors associated with self-medication in malaria treatment modality among selected parents and child care-givers in Nigeria. 2024.

[22] Chipwaza B, Mugasa JP, Mayumana I, Amuri M, Makungu C, Gwakisa PS. Self-medication with anti-malarials is a common practice in rural communities of Kilosa district in Tanzania despite the reported decline of malaria. Malar J. 2014;13:252. doi: 10.1186/1475-2875-13-252 24992941 PMC4087197

[23] Diiro GM, Affognon HD, Muriithi BW, Wanja SK, Mbogo C, Mutero C. The role of gender on malaria preventive behaviour among rural households in Kenya. Malar J. 2016;15:14. doi: 10.1186/s12936-015-1039-y 26738483 PMC4704398

[24] Mwita S, Meja O, Katabalo D, Richard C. Magnitude and factors associated with anti-malarial self-medication practice among residents of Kasulu Town Council, Kigoma-Tanzania. Afr Health Sci. 2019;19(3):2457–61. doi: 10.4314/ahs.v19i3.20 32127817 PMC7040277

[25] Negatu GA, Abebe GA, Yalew WG. Prevalence of Malaria and associated factors among malaria-suspected patients attending hamusit health center, Northwest Ethiopia: a cross-sectional study. J Parasitol Res. 2022;2022:1306049. doi: 10.1155/2022/1306049 35360675 PMC8964168

[26] Tairou F, Nawaz S, Tahita MC, Herrera S, Faye B, Tine RCK. Malaria prevention knowledge, attitudes, and practices (KAP) among adolescents living in an area of persistent transmission in Senegal: results from a cross-sectional study. PLoS One. 2022;17(12):e0274656. doi: 10.1371/journal.pone.0274656 36454893 PMC9714833

[27] Kojom LPF, Ntoumba AA, Nyabeyeu Nyabeyeu H, Bunda Wepnje G, Tonga C, Lehman LG. Prevalence, patterns and predictors of self-medication with anti-malarial drugs among Cameroonian mothers during a recent illness episode. J Med Biomed Sci. 2018;7(1):29–39. doi: 10.4314/jmbs.v7i1.4

[28] Susiku NG, Jacobs C, Zgambo J, Kaonga P, Sitali L. Determinants of self-treatment with antimalarials in Ndola district, Zambia: a cross-sectional study. medRxiv. 2024;2024:2024.09.02.24312958.

[29] Chuma J, Okungu V, Molyneux C. Barriers to prompt and effective malaria treatment among the poorest population in Kenya. Malar J. 2010;9:144. doi: 10.1186/1475-2875-9-144 20507555 PMC2892503

[30] Haule A, Mugashe H, Marwa K, Kapesa A, Hamasaki K, Mwita S. Self-medication practice with antimalarials and associated factors among undergraduate health science students in North Western - Tanzania: a cross-sectional study. EASci. 2023;5(1):73–80. doi: 10.24248/easci.v5i1.77

[31] Ruebush TK, Kern MK, Campbell CC, Oloo AJ. Self-treatment of malaria in a rural area of western Kenya. Bull World Health Organ. 1995;73(2):229–36. 7743595 PMC2486763

[32] Okeke TA, Uzochukwu BSC, Okafor HU. An in-depth study of patent medicine sellers’ perspectives on malaria in a rural Nigerian community. Malar J. 2006;5:97. doi: 10.1186/1475-2875-5-97 17078875 PMC1635422

[33] Ocan M, Bwanga F, Bbosa GS, Bagenda D, Waako P, Ogwal-Okeng J, et al. Patterns and predictors of self-medication in northern Uganda. PLoS One. 2014;9(3):e92323. doi: 10.1371/journal.pone.0092323 24658124 PMC3962384

[34] McCombie SC. Self-treatment for malaria: the evidence and methodological issues. Health Policy Plan. 2002;17(4):333–44. doi: 10.1093/heapol/17.4.333 12424205

[35] Asante FA, Asenso-Okyere K. Economic burden of malaria in Ghana. World Health Organization (WHO); 2003.

[36] Bayou FD, Arefaynie M, Tsega Y, Endawkie A, Kebede SD, Kebede N, et al. Effect of community based health insurance on healthcare services utilization in Ethiopia: a systematic review and meta-analysis. BMC Health Serv Res. 2024;24(1):1188. doi: 10.1186/s12913-024-11617-5 39369193 PMC11456236

[37] Fetene SM, Mengistu MY, Aschalew AY. Effectiveness and impact of community-based health insurance on health service utilization in northwest Ethiopia: a quasi-experimental evaluation. Front Public Health. 2023;11:1078462. doi: 10.3389/fpubh.2023.1078462 38026288 PMC10679351

[38] Bell G, Macarayan EK, Ratcliffe H, Kim J-H, Otupiri E, Lipsitz S, et al. Assessment of bypass of the nearest primary health care facility among women in Ghana. JAMA Netw Open. 2020;3(8):e2012552. doi: 10.1001/jamanetworkopen.2020.12552 32785634 PMC7424402

[39] Okunola O, Aroke A, Okunola G. Correlates of self-medication practices for malaria illness to under-five children in southwestern Nigeria. 2024.

